# The complete mitochondrial genome of the citrus red mite *Panonychus citri *(Acari: Tetranychidae): high genome rearrangement and extremely truncated tRNAs

**DOI:** 10.1186/1471-2164-11-597

**Published:** 2010-10-23

**Authors:** Ming-Long Yuan, Dan-Dan Wei, Bao-Jun Wang, Wei Dou, Jin-Jun Wang

**Affiliations:** 1 Key Laboratory of Entomology and Pest Control Engineering, College of Plant Protection, Southwest University, Chongqing 400716, China

## Abstract

**Background:**

The family Tetranychidae (Chelicerata: Acari) includes ~1200 species, many of which are of agronomic importance. To date, mitochondrial genomes of only two Tetranychidae species have been sequenced, and it has been found that these two mitochondrial genomes are characterized by many unusual features in genome organization and structure such as gene order and nucleotide frequency. The scarcity of available sequence data has greatly impeded evolutionary studies in Acari (mites and ticks). Information on Tetranychidae mitochondrial genomes is quite important for phylogenetic evaluation and population genetics, as well as the molecular evolution of functional genes such as acaricide-resistance genes. In this study, we sequenced the complete mitochondrial genome of *Panonychus citri *(Family Tetranychidae), a worldwide citrus pest, and provide a comparison to other Acari.

**Results:**

The mitochondrial genome of *P. citri *is a typical circular molecule of 13,077 bp, and contains the complete set of 37 genes that are usually found in metazoans. This is the smallest mitochondrial genome within all sequenced Acari and other Chelicerata, primarily due to the significant size reduction of protein coding genes (PCGs), a large rRNA gene, and the A + T-rich region. The mitochondrial gene order for *P. citri *is the same as those for *P. ulmi *and *Tetranychus urticae*, but distinctly different from other Acari by a series of gene translocations and/or inversions. The majority of the *P. citri *mitochondrial genome has a high A + T content (85.28%), which is also reflected by AT-rich codons being used more frequently, but exhibits a positive GC-skew (0.03). The Acari mitochondrial *nad1 *exhibits a faster amino acid substitution rate than other genes, and the variation of nucleotide substitution patterns of PCGs is significantly correlated with the G + C content. Most tRNA genes of *P. citri *are extremely truncated and atypical (44-65, 54.1 ± 4.1 bp), lacking either the T- or D-arm, as found in *P. ulmi*, *T. urticae*, and other Acariform mites.

**Conclusions:**

The *P. citri *mitochondrial gene order is markedly different from those of other chelicerates, but is conserved within the family Tetranychidae indicating that high rearrangements have occurred after Tetranychidae diverged from other Acari. Comparative analyses suggest that the genome size, gene order, gene content, codon usage, and base composition are strongly variable among Acari mitochondrial genomes. While extremely small and unusual tRNA genes seem to be common for Acariform mites, further experimental evidence is needed.

## Background

The family Tetranychidae (spider mites) (Chelicerata: Acari) includes ~1200 species, many of which are of agronomic importance [1], such as *Tetranychus urticae*, *Panonychus citri*, and *P. ulmi*; the former is a worldwide pest of many plant species including several economically important agricultural crops, while the latter two are important fruit plant (e.g., apple and citrus) pests. Spider mites are often difficult to manage because of their ability to rapidly develop resistance to various acaricides [2]. It has been reported that the resistance of *T. urticae *to the acaricide bifenazate is highly correlated with the remarkable mutations in the mitochondrial encoded cytochrome b (*cob*) [3]. In addition, several acaricides, such as acequinocyl, fluacrypyrim [4], and METI-acaricides (mitochondrial electron transfer inhibitors, e.g., fenpyroximate and pyridaben) [5], which are now in widespread use globally, target mitochondrial proteins. Unravelling and comparing mitochondrial genomes of spider mites will not only increase our understanding of the molecular evolution of acaricide-resistance genes, but also help to develop new acaricides uniquely targeting mitochondrial genes.

During the last 10 years, arthropod mitochondrial genomes have been extensively sequenced due to the improvements of genomic technologies and the interest in mitochondrial genome organization and evolution [6]. Mitochondrial genome sequences not only contain more information than the shorter sequences of single genes, but also provide larger data sets of genome-level features such as gene rearrangements and RNA secondary structures [6-8]. To date, the complete mitochondrial genomes have been sequenced from 25 Acari species. Among these, 14 species belong to the superorder of Parasitiformes while the remaining 11 species belong to the superorder of Acariformes. Most mitochondrial genomes are about 15 kb circular molecules and encode 37 genes including 13 protein-coding genes (PCGs), two rRNA genes (rRNAs), and 22 tRNA genes (tRNAs), which is typical of Metazoa [9]. However, the mitochondrial genomes of *Leptotrombidium pallidum *and *Metaseiulus occidentalis *possess duplicated genes [10,11], and the mtDNA of the sexual oribatid mite *Steganacarus magnus *lacks 16 tRNA genes [12]. In addition, gene rearrangement, reverse base composition, and atypical tRNA genes are frequently present in Acari, especially for the Acariformes, such as in the mite genera *Leptotrombidium *[10,13], and *Dermatophagoides *[14,15], and also in the mite species *P. ulmi *and *T. urticae *[3].

Generally, mitochondrial gene content and gene order are highly conserved at the lower taxonomic rank (i.e. family and genus), but gene content variation and arrangement have reported for the genera *Leptotrombidium *[10,13] and *Dermatophagoides *[14,15]. In addition, the inference of mite mitochondrial tRNA genes may be extremely difficult and even error prone, especially when comparative data is absent [15]. Thus, more mitochondrial genomes from closely related species will improve the accuracy of annotations for mitochondrial genomes, which will also greatly improve our understanding of molecular evolution and phylogenetic relationships [16-19]. In this study, we sequenced and analyzed the complete mitochondrial genome of the citrus red mite *P. citri *(Family Tetranychidae), an important citrus pest with a worldwide distribution, and provide a comparison to other Acari.

## Results and discussion

### Genome content and organization

**Figure 1:**
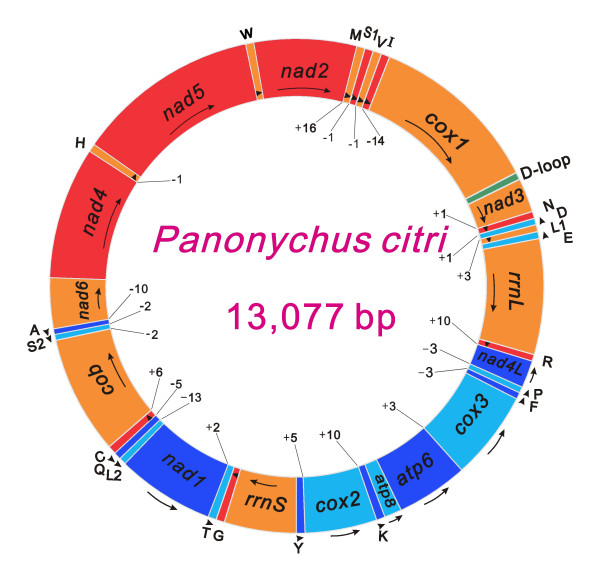
**Map of the mitochondrial genome of *Panonychus citri***. Genes coded in the J-strand (clockwise) are red or orange colored. Genes coded in the N-strand (counterclockwise) are blue or cyan colored. The D-loop (putative control region) is given in green. Alternation of colors was applied for clarity. Protein coding and ribosomal genes are shown with standard abbreviations. Genes for tRNA genes are abbreviated by a single letter, with S1 = AGN, S2 = UCN, L1 = CUN, and L2 = UUR. Numbers at gene junctions indicate the length of small non-coding regions where negative numbers indicate overlap between genes.

**Figure 2:**
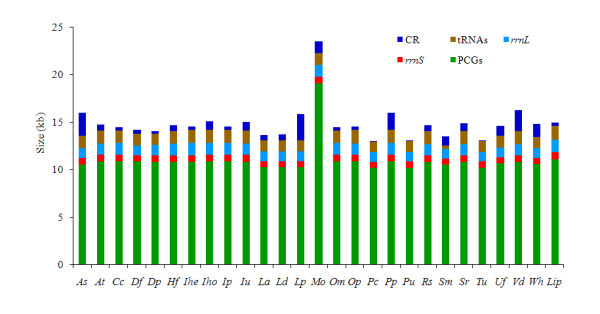
**The size of PCGs, *rrnL, rrnS*, and CR among Acari mitochondrial genomes**. Species are abbreviated as following: *As*, *Ascoschoengastia *sp.; *At*, *Amblyomma triguttatum*; *Cc*, *Carios capensis*; *Df*, *Dermatophagoides farinae*; *Dp*, *Dermatophagoides pteronyssinus*; *Hf*, *Haemaphysalis flava*; *Ihe*, *Ixodes hexagonus*; *Iho*, *Ixodes holocyclus*; *Ip*, *Ixodes persulcatus*; *Iu*, *Ixodes uriae*; *La*, *Leptotrombidium akamushi*; *Ld*, *Leptotrombidium deliense*; *Lp*, *Leptotrombidium pallidum*; *Lip*, *Limulus polyphemus*; *Mo*, *Metaseiulus occidentalis*; *Om*, *Ornithodoros moubata*; *Op*, *Ornithodoros porcinus*; *Pc*, *Panonychus citri*; *Pp*, *Phytoseiulus persimilis*; *Pu*, *Panonychus ulmi*; *Rs*, *Rhipicephalus sanguineus*; *Sm*, *Steganacarus magnus*; *Sr*, *Stylochyrus rarior*; *Tu*, *Tetranychus urticae*; *Ud*, *Unionicola foili*; *Vd*, *Varroa destructor*; *Wh*, *Walchia hayashii*.

The mitochondrial genome of *P. citri *is a typical circular DNA molecule of 13,077 bp in length (GenBank: HM189212, Figure 1, Additional File 1). This genome is only 2 bp larger than that of another *P. citri *strain (13,075 bp) [20] and slightly smaller than those of the same-family species *T. urticae *(13,103 bp) [3] and *P. ulmi *(13,115 bp) (Additional file 2). To date, the mitochondrial genome of *P. citri *is the smallest within all sequenced Acari and other Chelicerate genomes available in the GenBank (status August 20, 2010). Its relatively small size is primarily due to the significant size reduction of PCGs, *rrnL*, and the putative control region (A + T-rich region in arthropods) in comparison with other Acari (Figure 2). The mitochondrial genome of *P. citri *contains all 37 genes (13 PCGs, 22 tRNA genes, and two rRNA genes) which are typically present in metazoan mitochondrial genomes [9]. Among these, 20 genes (seven PCGs, two rRNA genes and 11 tRNA genes) are located on the majority strand (J-strand) and the others on the minority strand (N-strand) (Figure 1, Additional File 1). Gene overlaps have been found at 11 gene junctions and have involved a total of 55 bp; the longest overlap (14 bp) exists between *trnV *and *trnI*. In addition to the large non-coding region, several small non-coding intergenic spacers are present in the *P. citri *mitochondrial genome and are spread over ten positions, ranging in size from 1 to 16 bp.

The largest non-coding region, which presumably functions as the mitochondrial control region, is 57 bp long and is present between *cox1 *and *nad3 *(Figure 1, Additional File 1). This region is completely comprised of adenines and thymines (A + T-rich region) and is the third smallest among all sequenced mitochondrial genomes within Acari; only those of *P. ulmi *(55 bp) and *T. urticae *(44 bp) are slightly smaller (Figure 2). The maximum size difference found in the A + T-rich regions across all sequenced Acari mitochondrial genomes is 2,756 bp, indicating that strong size variation among Acari mitochondrial genomes is significantly correlated to the A + T-rich regions (Figure 2). This result is concordant with previous findings from other chelicerates [21] and insects [22-24]. In fact, the A + T-rich region has been identified as the source of size variation in the entire mitochondrial genome, usually due to the presence of a variable copy number of repetitive elements [25]. The relative location of the A + T-rich region also varies greatly among Acari with the ancestral pattern of arthropods being between *rrnS *and *trnI *[9].

**Figure 3:**
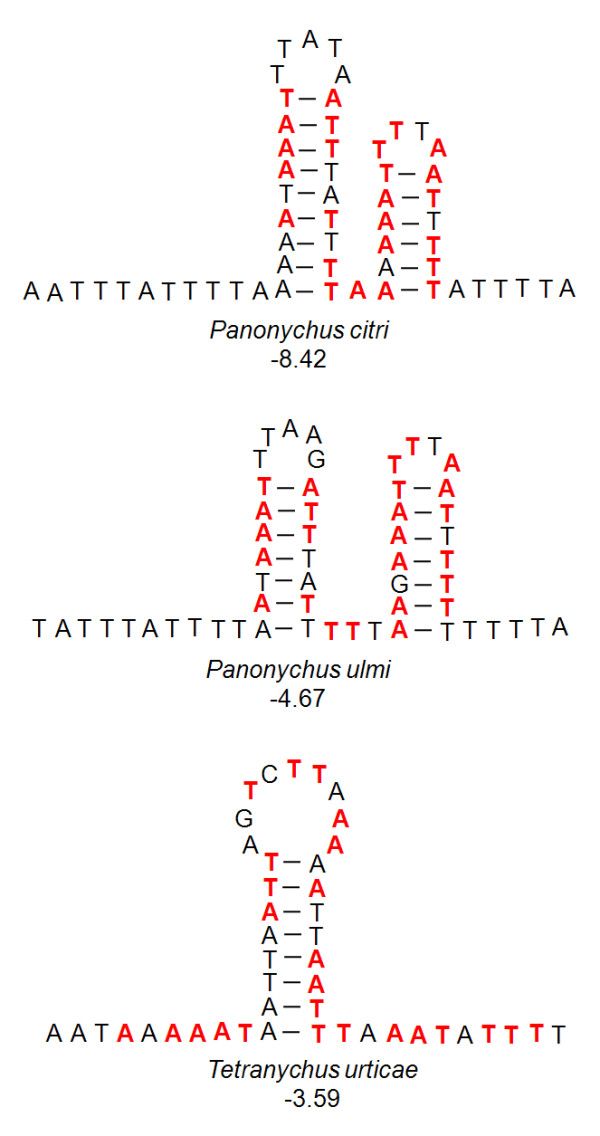
**The putative stem-loop secondary structures of A + T-rich region in the three spider mites *Panonychus citri, P. ulmi*, and *Tetranychus urticae*. **The secondary structures are constructed using Mfold Server [67], and free energy values (kcal/mol) are shown below each structure. The sequences conserved among the three spider mites are marked with red color.

Previous studies of insect A + T-rich region have identified extensive conserved sequence stretches and hypothetical secondary structure features, which are supposed to be responsible for the control of replication and transcription of the mtDNA [26-29]. In particular, one of the most conserved sequence stretches discovered in hexapod A + T-rich region seems to be an array of thymines (T-stretches), which are important signalling sites necessary for the initiation of the replication process. In the J-strand of *P. citri *mitochondrial genome, a short T-stretch (4 bp) has been found six nucleotides away from *cox1*, while a slightly longer T-stretch (6 bp) has been recognized in the N-strand 17 nucleotides away from *nad3 *(Figure 3). Similar T-stretch (4 bp) is also found in the J-strand of *P. ulmi *A + T-rich region, but no T-stretch can be found in the N-strand of *P. ulmi *and on both strand of *T. urticae *(Figure 3). It has been shown that the T-stretches vary in length, e.g., 4-10 nucleotides in the collembolan species, and >10 bp in holometabola [28,29]. While the minimum length of T-stretch is not known, long T-stretches (>10 bp) seem to be indispensable for the replication initiation of mtDNA [28]. Therefore, other sequences instead of the T-stretches may provide essential signals for the replication initiation of mtDNA in the species that do not possess the long T-stretches, e.g., *Locusta migratoria *[28]. However, the A + T-rich regions are highly variable among *P. citri*, *P. ulmi *and *T. urticae *(75.0% sequence identity), and only several short conserved sequences can be recognized (Figure 3). On the other hand, the stem-loop secondary structure in the A + T-rich region may also be involved in the initiation of the replication of animal mtDNA [30]. The A + T-rich region of the three spider mites can be folded into stable stem-loop secondary structures (Figure 3): two stem-loop structures are present in both *P. citri *and *P. ulmi*, whereas only one is found in *T. urticae*. It has been previously suggested that the sequence flanking the stem-loop structure is highly conserved among arthropods: a TATA motif in the 5'-flanking sequence and a GA(A)T motif in the 3'-flanking sequence [30]. Both motifs are presumed to have a functional significance in the replication and transcription of the mtDNA [30-32], but are not present in the A + T-rich regions of *P. citri*, *P. ulmi*, and *T. urticae *(Figure 3).

**Figure 4:**
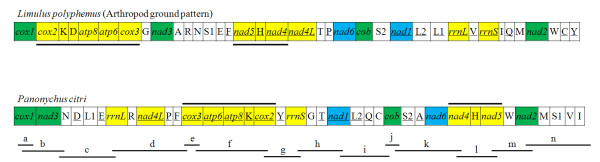
**Gene rearrangements of the *Panonychus citri *mitochondrial genome**. Only protein coding genes (PCGs) and ribosomal RNA genes (rRNAs) are marked, whereas transfer RNA genes (tRNAs) are not denoted because they are highly complex. Green boxes represent genes with the same relative position as in the arthropod ground pattern, *Limulus polyphemus*. Blue color indicates translocations, only, whereas yellow color denotes translocations and inversions, combined. Horizontal lines combine adjacent genes, which are probably subject to a joint inversion. tRNA genes are abbreviated using the one-letter amino acid code, with L1 = CUN; L2 = UUR; S1 = AGN; S2 = UCN. All genes are transcribed from left to right except those underlined to indicate an opposite transcriptional orientation. Genes are not drawn to scale and non-coding regions are not shown. PCR fragments (a-n) used to amplify the whole genome are also shown.

The gene order of the *P. citri *mitochondrial genome is identical to those of *P. ulmi *and *T. urticae *(the *trnK *of *P. ulmi *was misannotated on the opposite strand compared to *P. citri*, see below), but differs markedly from those of all other known Acari and chelicerates (Figure 1, Figure 4, Additional file 3, also see [14] and [33] for overviews of gene arrangement within Chelicerata). Compared to *Limulus polyphemus*, which is considered the representative ground pattern for arthropod mitochondrial genomes [34,35], a series of gene rearrangements have occurred in the evolutionary history of *P. citri*; only nine of the 38 gene boundaries in *L. polyphemus *are conserved in *P. citri*. The most striking features are the inversions of two segments, which change the relative position and transcriptional orientation of six PCGs (*cox3*, *atp6*, *atp8*, *cox2*, *nad4*, and *nad5*) and two tRNA genes (*trnK *and *trnH*); *trnD *may invert before or after the rearrangement of the fragment *cox3*-*cox2*. All 22 tRNA genes and two rRNA genes are also severely rearranged, translocated and/or inverted, but do not show any particular patterns.

So far, the complete mitochondrial genomes of 26 species belonging to Acari have been sequenced and they exhibit great variation of gene order (Additional file 3). Among them, 7 of the 14 species belonging to the superorder of Parasitiformes (e.g. *Ixodes *spp.) share the same gene order as the ancestral chelicerates, while all of the 12 species belonging to the superorder of Acariformes are highly rearranged [14,33], suggesting that these gene arrangements within Acari are independently derived [33]. The rearrangement events (translocations and/or inversion) from other Acari (compared to *L. polyphemus*) do not seem to be more parsimonious to produce the gene arrangement of *P. citri *(Additional File 3). It is clear that the unique mitochondrial genome arrangement present in *P. citri*, *P. ulmi*, and *T. urticae *likely occurred after Tetranychidae diverged from other Acari, or after Acariformes split from Parasitiformes and Opilioacariformes, because this feature is not shared with any other Acari. Rearrangements of the mitochondrial genome should be relatively rare events at the evolutionary scale, and, therefore, provide a powerful tool to delimit deep divergences among some metazoan lineages [36]. However, the mitochondrial gene rearrangement phenomenon seems to occur frequently and independently in Acari mitochondrial genomes [8,33], possibly restricting the phylogenetic applications in recovering the evolutionary relationships between superorders within the Acari. On the other hand, gene order appears to be linked to taxonomic relatedness at the lower rank (i.e. genus, family). For example, the relationship between prostriate ticks and metastriate ticks can be distinguished by gene arrangements [31,37].

### Base composition and codon usage

**Figure 5:**
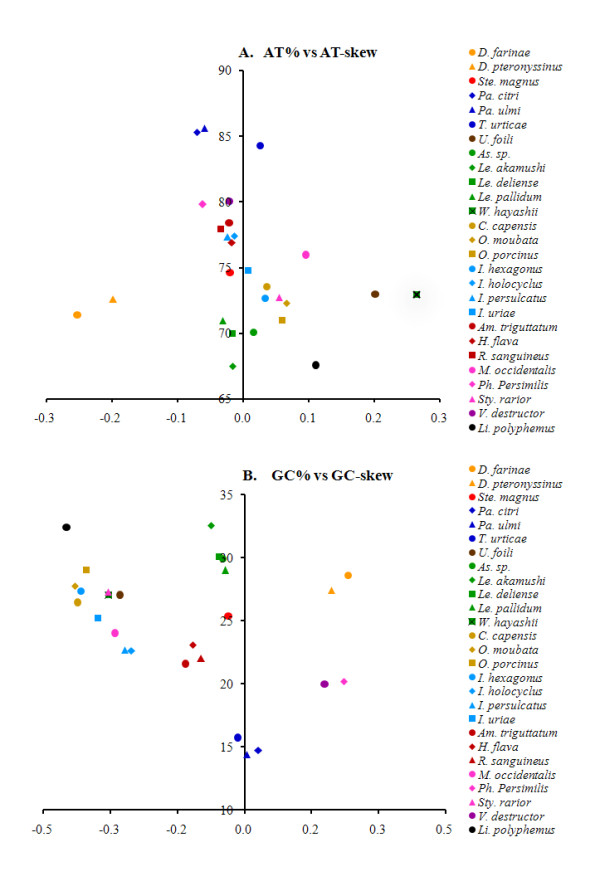
**AT% *vs *AT-skew and GC% *vs *GC-skew in the 26 Acari mitochondrial genomes**. Measured in bp percentage (Y-axis) and level of nucleotide skew (X-axis), following [48]. Values are calculated on J-strands for full length mitochondrial genomes. A, A + T% *vs *AT-skew; B, G + C% *vs *GC-skew. Genome sequence from *Limulus polyphemus *is also shown for a comparative analysis. See Additional file 11 for the GenBank accession numbers and full names of species.

As is the case in other Acari mitochondrial genome sequences, the nucleotide composition of the *P. citri *mtDNA is also biased toward A and T (J-strand: A = 39.62%, T = 45.66%, G = 7.58%, C = 7.14%; Additional file 1). A comparative analysis of A + T% *vs *AT-skew and G + C% *vs *GC-skew across all available complete mitochondrial genomes of Acari and *L. polyphemus *is shown in Figure 5. The overall A + T content (85.28%) of *P. citri *is similar to those of *P. ulmi *(85.60%) and *T. urticae *(84.27%), but much higher than the average A + T content of Acari mitochondrial genomes (75.34 ± 4.78%) as well as that of *L. polyphemus *(67.57%).

Metazoan mitochondrial genomes usually present a clear strand bias in nucleotide composition [38,39]. In detail, the J-strand is biased in favour of adenine and cytosine, while the N-strand consequently contains more thymine and guanine. The strand bias can be measured as AT- and GC-skews [40]. The average AT-skew of Acari mitochondrial genome is 0.000 ± 0.101, ranging from -0.253 in *Dermatophagoides farinae *to 0.264 in *Walchia hayashii*, whereas the *P. citri *mitochondrial genome exhibits a slight AT-skew (0.071) (Figure 5). The marked AT-skew values are shared by the mitochondrial genomes of *D. farinae *(-0.253), *D. pteronyssinus *(-0.199), *Unionicola foili *(0.201), and *W. hayashii *(0.264).

The average GC-skew of Acari mitochondrial genome is -0.126 ± 0.195 and most of GC-skew values are negative, similar to those typically found in most metazoan mitochondrial genomes. However, as has been reported for other arthropods [38,39,41-43], six mite species in four genera are characterized by a reversal of GC-skew: *Dermatophagoides *[14,15], *Phytoseiulus *[44], *Varroa *[45], and *Panonychus *(Figure 5). This reversal of the strand bias could be the result of inversion of the control region [38], which contains all initiation sites for replication and transcription of the mtDNA [46]. Therefore, an inversion of the control region is expected to produce a global reversal of asymmetric mutational constraints in the mtDNA, with time resulting in a complete reversal of strand compositional bias [38]. In the *P. citri *mtDNA most genes encoded on the J-strand show a positive GC-skew at the fourfold degenerate third codon position which is inverted to the common pattern and probably indicates a reversal of the control region (Additional file 1). Furthermore, this reverse strand bias may occur after the genus *Panonychus *split from the family Tetranychidae, because *T. urticae*, belonging to the same-family (Tetranychidae) with *P. citri *and *P. ulmi*, shows a usual GC-skew (negative), as found in most of Acari.

The analyses of the base composition at each codon position of the concatenated 13 PCGs of *P. citri *show that the third codon positions (92.9%) have an A + T content higher than that of the first (82.8%) and second (79.4%) codon positions, as has also been found in other sequenced Acari (Additional file 4). The relative synonymous codon frequencies (RSCU) reveal that codons harbouring A or T in the third position are always overused as compared to other synonymous codons in the *P. citri *mitochondrial genome (Table 1), as found in other sequenced Acari (Additional file 5) and other chelicerates [21,47]. Among 62 amino-acid encoding codons of invertebrate mitochondrial code [9], the *P. citri *mitochondrial genome uses 57 codons and never utilizes the five G + C rich codons. Furthermore, the four AT-rich codons TTT-Phe (15.3), TTA-Leu (11.4), ATT-Ile (10.6), and ATA-Met (10.8) are the most frequently used codons in the *P. citri *PCGs, (Table 1). Comparative analyses among other sequenced Acari also reveal a similar pattern with the exceptions of *D. farinae *and *S. magnus*, and the values range from 26.40% in *Leptotrombidium akamushi *to 48.83% in *P. ulmi *(Additional file 5). This noted difference in the four most frequently used codons between Acari seems to be directly linked to the overall A + T content (Additional file 6), as reported in other chelicerates and insects [47,48].

**Table 1:** Codon usage for the 13 mitochondrial proteins of *Panonychus citri*

AA	Codon	n	%	RSCU	AA	Codon	n	%	RSCU
Stop	UAA	0	0	0	Asn (N)	AAC	24	0.71	0.17
	UAG	0	0	0		AAU	262	7.76	1.83
Ala (A )	GCU	39	1.16	3.06	Pro (P)	CCU	53	1.57	3.21
	GCG	1	0.03	0.08		CCG	0	0	0
	GCC	1	0.03	0.08		CCC	4	0.12	0.24
	GCA	10	0.30	0.78		CCA	9	0.27	0.55
Cys (C)	UGU	16	0.47	1.68	Gln (Q)	CAG	2	0.06	0.14
	UGC	3	0.09	0.32		CAA	26	0.77	1.86
Asp (D)	GAU	38	1.13	1.73	Arg (R)	CGA	16	0.47	2.29
	GAC	6	0.18	0.27		CGC	0	0	0
Glu (E)	GAG	10	0.30	0.31		CGG	0	0	0
	GAA	55	1.63	1.69		CGU	12	0.36	1.71
Phe (F)	UUU	516	15.28	1.90	Ser1 (S)	AGC	8	0.24	0.22
	UUC	28	0.83	0.10		AGA	65	1.93	1.75
Gly (G)	GGU	30	0.89	1.29		AGG	0	0	0
	GGG	14	0.41	0.60		AGU	39	1.16	1.05
	GGC	2	0.06	0.09	Ser2 (S)	UCA	48	1.42	1.29
	GGA	47	1.39	2.02		UCC	8	0.24	0.22
His (H)	CAC	4	0.12	0.17		UCG	1	0.03	0.03
	CAU	42	1.24	1.83		UCU	129	3.82	3.46
Ile (I)	AUU	359	10.63	1.87	Thr (T)	ACA	35	1.04	1.46
	AUC	26	0.77	0.14		ACC	2	0.06	0.08
Lys (K)	AAA	150	4.44	1.86		ACG	2	0.06	0.08
	AAG	11	0.33	0.14		ACU	57	1.69	2.38
Leu1 (L1)	CUA	22	0.65	1.42	Val (V)	GUC	1	0.03	0.04
	CUC	0	0	0		GUG	2	0.06	0.08
	CUG	3	0.09	0.19		GUU	51	1.51	2.13
	CUU	37	1.10	2.39		GUA	42	1.24	1.75
Leu2 (L2)	UUA	385	11.40	1.86	Trp (W)	UGA	44	1.30	1.91
	UUG	28	0.83	0.14		UGG	2	0.06	0.09
Met (M)	AUG	29	0.86	0.15	Tyr (Y)	UAC	17	0.50	0.22
	AUA	363	10.75	1.85		UAU	140	4.15	1.78

A total of 3,376 codons for *P. citri *are analyzed, excluding the start and stop codons. AA, amino acid; RSCU, Relative synonymous codon usage; n = frequency of each codon. % = n/3376.

### Protein-coding genes

The total length of all the 13 PCGs is 10,196 bp, and accounts for 77.97% of the entire length of *P. citri *mitochondrial genome (Additional file 1). The overall A + T content of PCGs is 73.91%, ranging from 78.45% (*cox1*) to 92.06% (*nad6*). All the PCGs start with ATN codons, which is typical for metazoan mitochondria [49]: one (*cox1*) with ATC, three (*nad3*, *nad4L*, and *nad1*) with ATA, four (*cox2*, *cox3*, *atp6*, and *nad4*) with ATG, and the other five with ATT (Additional file 1). Eight PCGs terminate with the conventional stop codons TAA (*cox1*, *nad3*, *nad4L*, *atp6*, *cox2*, *nad2*, and *nad4*) or TAG (*cob*), whereas the remaining five have incomplete stop codons T. The presence of an incomplete stop codon is common in metazoan mitochondrial genomes [49] and these truncated stop codons are presumed to be completed via post-transcriptional polyadenylation [50]. A comparison of codon numbers across the sequenced Acari mitochondrial genomes shows that all PCGs are highly variable in length, covering a range from 3,389 bp in *P. citri *to 6,328 bp in *M. occidentalis *(Figure 2). Notably, the *M. occidentalis *mitochondrial genome has the largest number of codons within chelicerates due to the duplication of many PCGs, even though *nad3 *and *nad6 *are lost [11]. However, a recent study has found that the *M. occidentalis *mtDNA is smaller than originally suggested, and *nad3 *is not lacking but is in fact located between *nad4L *and *rrnS *[44]. *P. citri*, *P. ulmi*, and *T. urticae *have a reduction in mean content of 19.8, 19.8, and 19.0 codons per PCG, respectively, making them the three smallest mitochondrial genomes within Acari.

**Figure 6:**
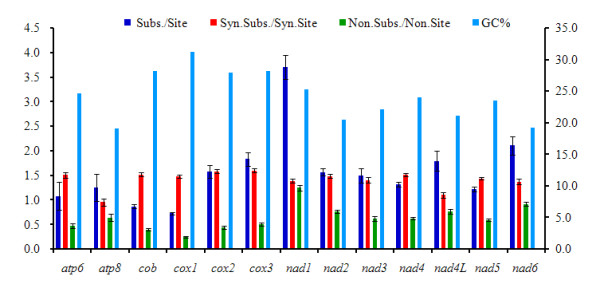
**Different evolutionary patterns among 13 Acari mitochondrial PCGs**. The left Y-axis provides the substitution rate of mitochondrial gene, while the right Y-axis provides the G + C content, according to [23]. Subs./Site, the nucleotide substitution number per site from the averaging over all sequence pairs of each gene, and the analyses were conducted using the Jukes-Cantor (Jukes and Cantor 1969) method. Syn.Subs./Syn.Site, synonymous nucleotide substitutions per synonymous site; Non.Subs./Non.Site, nonsynonymous nucleotide substitutions per nonsynonymous site; the analyses were conducted using the Kumar method [70]. The rate variation among sites was modelled with a gamma distribution estimated with the jModeltest [71]. The standard error estimates were obtained by a bootstrap procedure (500 replicates).

The 13 PCGs of *P. citri *were combined with those of other Acari to investigate the evolutionary patterns among PCGs in Acari. The nucleotide substitution rates per site greatly vary among genes (Figure 6). In this respect, *nad1 *shows the highest value (3.69 ± 0.24), followed by *nad6*, while *cox1 *appears to be the lowest. The number of synonymous substitutions per synonymous site (*Ks*) of *cox3 *is the highest, but its number of nonsynonymous substitutions per nonsynonymous site (*Ka*) is much lower, while this value for *nad1 *is the highest. Therefore, at the nucleotide and amino acid levels, *cox1 *is the slowest evolving protein, making it a useful marker for investigating phylogenetic relationships at higher taxonomic levels. However, a DNA barcoding approach based on *cox1 *sequence diversity has been utilized for identification of closely related species [51]. By contrast, due to the highest divergence, *nad1 *can be used as an effective molecular marker to analyze intraspecific relationships and reveal relationships between populations within the same Acari species. The *Ks *of each PCG has a relatively high value compared to *Ka*, which may indicate that these genes are evolving under purifying selection [16,52]. Notably, *nad1 *accumulates amino acid substitutions twice as fast as the Acari mitochondrial average, possibly implying positive selection acting at *nad1 *or due to relax selection. Furthermore, a negative correlation has been found between the *Ka*/*Ks *and the GC content of each PCG (R = -0.676, *P *= 0.011), indicating that the variation of GC content probably causes the different evolutionary patterns among genes [23].

### Transfer and ribosomal RNA genes

The two genes encoding the large and small rRNA subunits (*rrnL *and *rrnS*) are located between *trnE *and *trnR*, and between *trnY *and *trnG *(Figure 1, Additional file 1). Both genes are encoded on the J-strand, as in *P. ulmi*, *T. urticae *[3], *M. occidentalis *[11], and *Phytoseiulus persimilis *[44], and in the two genera *Dermatophagoides *[14,15] and *Leptotrombidium *(but *L. pallidum *has a duplicated *rrnL *gene on the N-strand) [10,13]. In contrast, in the mitochondrial genomes of other Acari and most species of chelicerates and arthropods, both rRNA genes are encoded on the N-strand [12,31,33,34,43,47,53,54]. We annotate the 5'- and 3'-ends of two rRNA genes as the first nucleotide downstream and upstream of corresponding tRNA genes, respectively, and the size of the *rrnL *and *rrnS *of *P. citri *are 989 bp and 648 bp long, respectively (Additional file 1). The A + T contents of *rrnL *and *rrnS *(86.66% and 87.54%, respectively) are similar to those of *P. ulmi *(86.76% and 88.01%, respectively) and *T. urticae *(85.27% and 85.91%, respectively), but much higher than the average value of Acari (78.58 ± 4.57% and 77.17 ± 5.08%, respectively). The boundaries of some tRNA genes are incorrectly delimited in *P. ulmi*, *T. urticae *and another strain of *P. citri *(see below). This aspect affects the boundaries of *rrnL *and *rrnS *in these taxa. Thus we re-annotate also these genes (Additional file 2). The size of *rrnL *and *rrnS *of *P. citri *is similar to those of another *P. citri *strain (992 bp and 650 bp), *P. ulmi *(983 bp and 659 bp) and *T. urticae *(992 bp and 645 bp). The size of *rrnL *of *P. citri *is the second shortest among all sequenced Acari mitochondrial genomes, whereas the size of *rrnS *is slightly larger than those of other Acariform mites (637.83 ± 27.70 bp), but are much shorter than those found in the Parasitiformes (702.57 ± 21.52 bp) (Figure 2).

**Figure 7:**
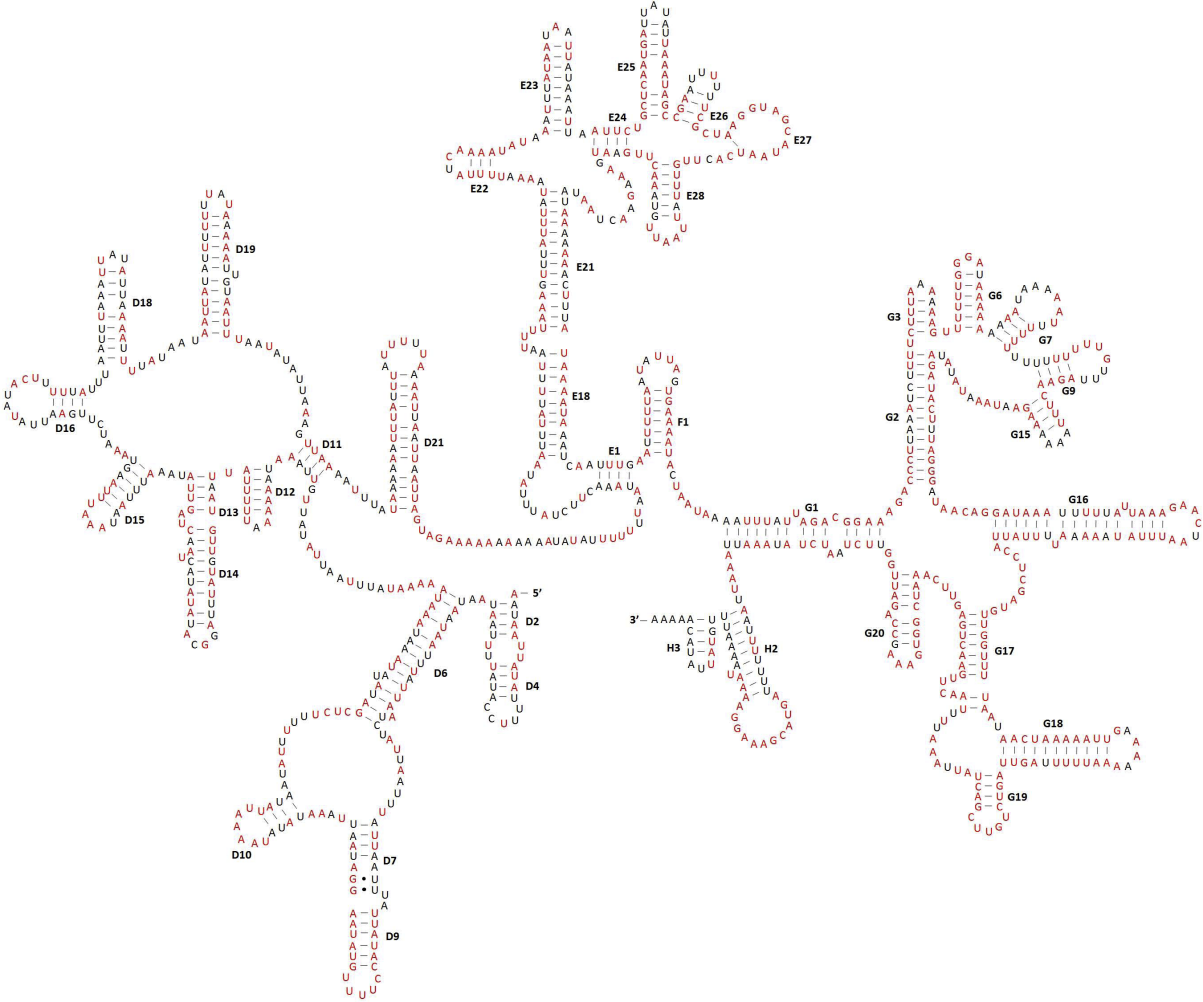
**Inferred secondary structure for the *rrnL *in *Panonychus citri*. **The helix numbering is after [72]. The nucleotides showing 100% identity among *P. citri*, *P. ulmi*, and *Tetranychus urticae *are marker with red color. Inferred Watson-Crick bonds are illustrated by lines, whereas GU bonds are illustrated by dots.

**Figure 8:**
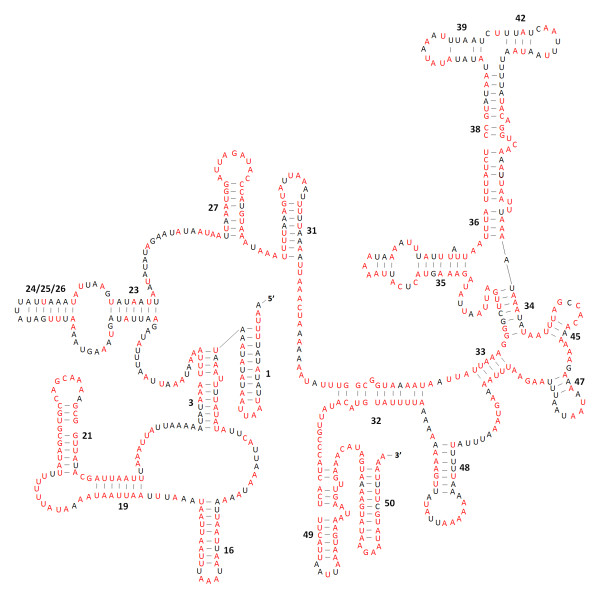
**Inferred secondary structure for the *rrnS *in *Panonychus citri*. **The helix numbering is after [73]. The nucleotides showing 100% identity among *P. citri*, *P. ulmi *and *Tetranychus urticae *are marker with red color. Inferred Watson-Crick bonds are illustrated by lines, whereas GU bonds are illustrated by dots.

The secondary structures of the two rRNA genes inferred for *P. citri *have similar stem-loop structures as those published for *L. pallidum *[13] and *D*. *pteronyssinus *[14] (Figure 7, Figure 8). All the helices of the *rrnL *found in *L. pallidum *are present in *P. citri*; however, three helices (C1, E2 and E19) found in *D*. *pteronyssinus *are not present in *P. citri*. The lack of helix C1 is largely due to the size reduction of the *P*. *citri rrnL *compared to the *D*. *pteronyssinus rrnL*, and the deleted nucleotides are mainly located at 5'-end. The helix H3 is similar in size to those of *L. pallidum *and *D*. *pteronyssinus*, but appears to be lost in *P. ulmi *and *T. urticae*, though the length of the three spider mite species is similar. Compared to the 5'-end, the 3'-end of *rrnL *structure is more conserved among *P. citri*, *P. ulmi*, and *T. urticae *(Figure 7), especially for the helices G16-G20. In addition, the loop region of helix H2 is highly conserved not only in Tetranychidae, but also in the other two mite genera *Leptotrombidium *[10,13] and *Dermatophagoides *[14,15]. Like in *L. pallidum *and *D*. *pteronyssinus*, one compound helix replaces three helices (24-25-26) of the *Drosophila yakuba rrnS *[13]. In the middle of *rrnS*, two helices (39 and 42) are present in *P. citri*, which is more similar to that of *S. magnus *[12], but only a compound helix (39-40-41-42) is present in *L. pallidum *and *D*. *pteronyssinus*. In addition, the helix 1 not present in *L. pallidum *is found in the *rrnS *of *P. citri*; this helix is also found in *D*. *pteronyssinus*. The most conserved sequences of *rrnS *among Tetranychidae are found in the helices 19, 21, 32, 33, 49, and 50 (Figure 8).

Out of 22 tRNA genes usually present in metazoan mitochondrial genomes, only 13 tRNA genes can be detected in the *P. citri *mitochondrial genome by tRNAscan-SE [55] and/or ARWEN [56]. The remaining nine tRNA genes (*trnN*, *trnD*, *trnP*, *trnY*, *trnS2*, *trnA*, *trnS1*, *trnV*, *trnI*) were identified by manually aligning unassigned sequences to known tRNA genes from *T. urticae *and *P. ulmi*, as suggested by Masta and Boore [43]. All 22 tRNA sequences were aligned with those of *P. ulmi *and *T. urticae*, and show high similarity among Tetranychidae, especially for the anticodon stem (Additional file 7). However, only eight tRNA genes (*trnF*, *trnY*, *trnT*, *trnL2*, *trnC*, *trnH*, *trnW*, and *trnM*) of both *P. ulmi *and *T. urticae *are correctly annotated on GenBank. Among the remaining tRNA genes, the boundaries of ten tRNA genes of another *P. citri *strain (*trnR*, *trnP*, *trnG*, *trnT*, *trnQ*, *trnC*, *trnA*, *trnS1*, *trnV*, and *trnI*) and *T. urticae *(*trnN*, *trnD*, *trnE*, *trnP*, *trnG*, *trnQ*, *trnS2*, *trnA*, *trnV*, and *trnI*), and 11 tRNA genes of *P. ulmi *(*trnN*, *trnL1*, *trnR*, *trnP*, *trnK*, *trnQ*, *trnS2*, *trnA*, *trnS1*, *trnV*, and *trnI*) were incorrect on GenBank (Additional file 2), because most of them appear to lack the sequences to form the canonical amino acid acceptor stem (seven nucleotides) or at least one D- or T-arm (Additional file 8). The *trnK *of *P. ulmi *is misannotated on the J-strand, but in this location an unpaired anticodon stem is found. In fact, the majority of the nucleotides of *trnK *among Tetranychidae are highly conserved (Additional file 7), and only when *trnK *of *P. ulmi *is on the N-strand, as in *P. citri *and *T. urticae*, can canonical secondary structure of tRNA genes be found (Additional file 8). Therefore, we re-annotate these tRNA genes to incorporate the conserved structural features of tRNA genes (e.g., the possession of seven nucleotides in the amino acid acceptor stem, and at least one T- or D-arm) (Additional file 2), and present their secondary structures in Additional file 8.

**Figure 9:**
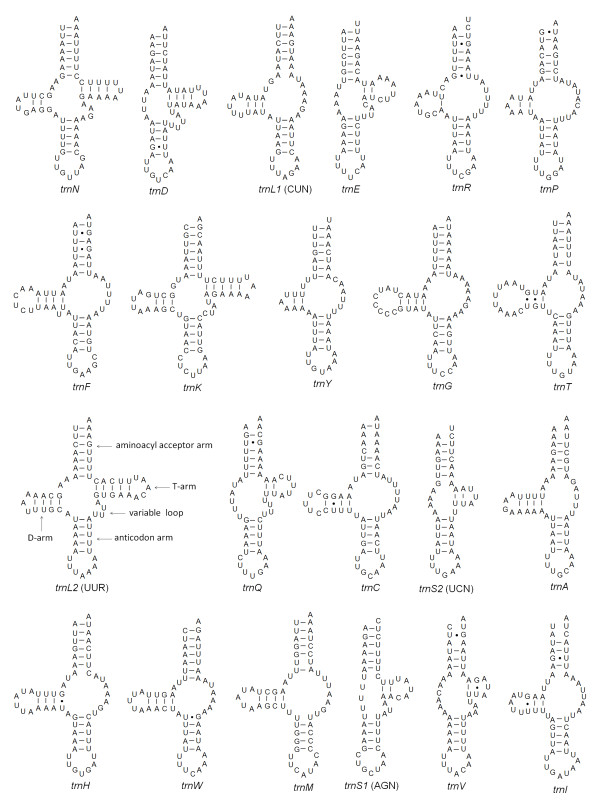
**Putative secondary structures of the 22 tRNA genes identified in the mitochondrial genome of *Panonychus citri***. tRNA genes are shown in the order of occurrence in the mitochondrial genome starting from *cox1*. Bars indicate Watson-Crick base pairings, and dots between G and U pairs mark canonical base pairings appearing in RNA.

Eleven tRNA genes overlap with adjacent genes in a total of 55 bp, as in another *P. citri *strain, whereas ten and nine gene overlaps are present in *P. ulmi *and *T. urticae*, respectively (Additional file 2). The most truncated tRNA genes are *trnQ*, *trnA*, and *trnI *in *P. citri*, whose sequences overlap the *trnL2*, *nad6 *and *trnV *on the same or opposite strand for 13, 10, and 14 nucleotides, respectively (Additional file 1). The similar situations are found in *P. ulmi *and *T. urticae *(Additional file 2). The similar amount of gene overlap on the same strand has been reported for the spider *Habronattus oregonensis *[43] and the house dust mite *D. pteronyssinus *[14]. Furthermore, if the three tRNA genes (*trnQ*, *trnA*, and *trnI*) do not overlap adjacent genes, they lack the sequences to form either the usual amino acid acceptor stem or at least one D- or T-arm. In addition, the nucleotides present in the anticodon arm of the three tRNA genes are highly conserved among Tetranychidae (Additional file 7). Other gene overlaps found in tRNA genes of Tetranychidae are not more than five nucleotides (Additional file 2). The average size (54.1 ± 4.1 bp) of tRNA genes found in *P. citri *is similar to those of *P. ulmi *(55.1 ± 4.5) and *T. urticae *(54.1 ± 3.6 bp), reducing the potential of tRNA genes to form a typical metazoan cloverleaf structure (Figure 9). In fact, the average size of tRNA genes of Acariformes (mean = 54.8 ± 1.0 bp) is considerably shorter with respect to those of all species of Parasitiformes (mean = 62.0 ± 1.3 bp) as well as *L. polyphemus *(mean = 66.3 ± 2.5 bp, Additional file 9), indicating that atypical tRNA genes may be a common phenomenon in Acariformes.

Out of 22 tRNA genes, only three tRNA genes (*trnN*, *trnL2*, and *trnK*) can potentially fold into a typical cloverleaf structure, whereas all the remaining 19 tRNA genes appear to lack the sequence to code the D- or T-arm (Figure 9), as found in *P. ulmi *and *T. urticae *(Additional file 8). In at least 13 tRNA genes, the T-arm has been substituted by a loop of variable size (TV-replacement), whereas another six tRNA genes (*trnD*, *trnE*, *trnQ*, *trnS1*, *trnS2*, and *trnV*) show a D-replacement loop instead of the D-arm (Figure 9). With the exception of *trnP*, which seems to be lost the D-arm in *P. ulmi*, all tRNA genes have similar secondary structures among Tetranychidae (Figure 9, Additional file 8). The loss of the D-arm in *trnS1 *(AGN) has been considered a typical feature of metazoan mitochondrial genomes [49]. The absence of the T-arm seems to be a common feature for the tRNA genes of chelicerates from the orders of the Araneae, Scorpiones, Thelyphonida, and Acariform Acari [10,13-15,43,57], whereas other orders (Amblypygi, Opiliones, Solifugae, and Parasitiform Acari) possess typical metazoan cloverleaf tRNAs [57]. On the other hand, the loss of the D-arm of tRNA genes is an uncommon circumstance but has been reported for a few of mitochondrial tRNA genes from chelicerates, including the scorpion *Centruroides limpidus *[58], the sea spiders *Nymphon gracile *[47] and *Achelia bituberculata *[21], and five mites belonging to two genera *Leptotrombidium *[10,13] and *Dermatophagoides *[15]. It has been shown that in the nematode *Ascaris suum *the tRNA genes that lack either the D- or T-arm are functional [59], but functional tRNA genes that lack both the D- and T-arms have not been found before. However, tRNA genes that lack both T- and D-arms have been reported for the sea spider *A. bituberculata *(*trnA*) [21] and the scorpion *C. limpidus *(*trnQ *and *trnS1*) [58]. In the American house dust mite *D. farinae*, some tRNA genes (e.g. *trnA*) lacking the D-arm have a small (2-3 bp) and thermodynamically unstable T-arm, suggesting that these tRNA genes may have lost both D- and T-arms in reality [15]. In this study, we also found that some inferred T-arms (e.g., *trnS2*, *trnV*) or D-arms (e.g., *trnY*, *trnR*, *trnP*) were short (Figure 9). In particular, the inferred D-arm of *trnY *in *P. ulmi *and *T. urticae *(only one bp) is shorter than in *P. citri*, whereas the D-arm of *trnP *is lost in *P*. *ulmi*, casting doubt on their identity as D-arms. Therefore, further experiments are needed to investigate whether these truly tRNA genes lack both D- and T-arms and if so, whether they are functional.

Twenty of 22 tRNA genes have a five bp well-paired anticodon stem, and the remaining two tRNA genes (*trnM *and *trnS1*) have a single mismatch within this stem. All tRNA genes, but *trnI*, have the seven canonical nucleotides in the anticodon loop, whereas *trnI *has eight nucleotides in this region, which is also found for *trnI *of *P. ulmi *and *T. urticae *(Additional file 8). These noncanonical anticodon loops are not common, but have also been reported for the house dust mite *D. pteronyssinus *(*trnL2*) [14], the scorpion *Mesobuthus gibbosus *(*trnH *and *trnN*) [58], and the wild two-humped *Camelus bactrianus ferus *(*trnS1*) [60]. Only nine of the 22 tRNA genes have a completely matched seven bp aminoacyl acceptor stem (*trnN*, *trnD*, *trnE*, *trnG*, *trnL2*, *trnK*, *trnF*, *trnW*, and *trnY*), while the remaining 13 tRNA genes have 1-3 bp mismatches in this stem. This type of a mis-paired acceptor stem seems to be a common phenomenon for tRNA genes of chelicerates (e.g., Araneae [42,43], Acari [14,15], Scorpiones [58]), and a posttranscriptional RNA editing mechanism has been proposed to maintain function of these tRNA genes [43,61].

## Conclusions

We sequenced the complete mitochondrial genome of the spider mite, *P. citri *(Family Tetranychidae). This mitochondrial genome in size is similar to those of other spider mites, *P. ulmi *and *T. urticae*, and is the smallest among all sequenced Acari and other Chelicerate genomes. The gene order of the *P. citri *mtDNA is identical to those of *P. ulmi *and *T. urticae*, but markedly differs from those of other chelicerates by a large number of gene inversions and/or translocations, suggesting that rearrangements occurred after Tetranychidae diverged from other Acari. Comparative analyses among Acari mitochondrial genomes show that the genome size, gene order, gene content, codon usage, and base composition are strongly variable. The Acari mitochondrial *nad1 *exhibits a faster amino acid substitution rate than the average, and the G + C content variation causes the different evolutionary patterns among genes of Acari mitochondrial genomes. Most tRNA genes present in *P. citri *are minimal and atypical, lacking the D- or T-arm, as found in *P. ulmi *and *T. urticae*. While extremely short and unusual tRNA genes seem to be common for Acariform mites, further experimental evidence is needed.

## Methods

### Samples and DNA extraction

Female adult of *P. citri *was collected on Trifoliate orange from Citrus Research Institute of Chinese Academy of Agricultural Sciences, Chongqing, China. Total DNA was extracted from about 800 females using a CTAB-based protocol [62] and stored at -20°C.

### PCR amplification and sequencing

Fourteen pairs of PCR primers were employed to amplify overlapping segments of the entire mitochondrial genome of *P. citri*. Initially, *cox1 *and *cob *genes were amplified using the primers COI-F/R [63,64] and CB3-F/CB4-R [65], respectively. Four perfectly matching primers, namely cox1-R1, cox1-F4, cob-R3 and cob-F3, were designed on the basis of the sequence information from *cox1 *and *cob*. Other primers were designed based on the conserved nucleotide sequences in *T. urticae *[3] and *P. ulmi *(GenBank: NC_012571). A full of list of primers as well as PCR conditions are presented in Additional file 10. All PCR products were separated by electrophoresis on a 1% agarose gel, purified with DNA Gel Purification Kit (Watson, Shanghai), and cloned into the pGEM-T vector (Promega, USA). After heat-shock transformation of *Escherichia coli *(*Trans5α*, Beijing TransGen Biotech) cells, the positive recombinant clone with an insert was sequenced with M13 primers on both strands.

### Sequence assembly, annotation and analysis

Sequence data were assembled using SeqMan software (DNAStar, Inc.). Protein coding genes (PCGs) were identified by ORF Finder implemented at the NCBI website with the invertebrate mitochondrial genetic codes and by comparison with the published Acari mitochondrial sequences with Clustal W 2.0 [66]. Two large non-protein-coding regions were candidates for the rRNA genes (*rrnL *and *rrnS*). The boundaries were determined based on alignments and secondary structures of rRNA sequences of other mite species. The tRNA genes were identified by their cloverleaf secondary structure using tRNAscan-SE 1.21 [55] and ARWEN [56]. For tRNAscan-SE the following parameters were changed: Search Mode = "EufindtRNA-Cove", Source = "Nematode Mito", Genetic Code = "Invertebrate Mito" and Cove score cutoff = 0.1. ARWEN was run with default parameters. Other tRNA genes were identified by aligning to known tRNA genes from *P. ulmi *and *T. urticae *[43]. The secondary structure models for two rRNA genes of *P. citri *were constructed by comparison with the published rRNA secondary structures for *L. pallidum *[13], *D. pteronyssinus *[14], and *S. magnus *[12]. The secondary structure of A + T-rich region (putative control region) was constructed using Mfold Server [67]. The complete mitochondrial genome sequence of *P. citri *has been deposited in the GenBank database under the accession number HM189212. The base composition, codon usage, and nucleotide substitution were analyzed with Mega 4.0 [68] and DAMBE 5.0.59 [69]. Mitochondrial genome sequences from other Acari and the horseshoe crab *L. polyphemus *were obtained for comparative analyses (see Additional file 11 for GenBank accession numbers).

## Abbreviations

PCGs: protein-coding genes; *atp6 *and *atp8*: genes for the ATPase subunits 6 and 8; *cox1*-*cox3*: genes for cytochrome C oxidase subunits I-III; *cob*: a gene for apocytochrome b; *nad1*-*nad6 *and *nad4L*: genes for NADH dehydrogenase subunits 1-6 and 4L; *rrnL*: large (16S) rRNA subunit (gene); *rrnS*: small (12S) rRNA subunit (gene); *trnX *(where X is replaced by one letter amino acid code of the corresponding amino acid), transfer RNA.

## Authors' contributions

MLY designed the experiments, carried out the data analyses and drafted the manuscript, tables and figures. DDW, BJW, and WD participated in the experiments. JJW supervised this study, contributed to the design of the study and drafting the manuscript. All authors read and approved the final manuscript.

## Supplementary Material

Additional file 1 **Summary of the mitochondrial genome of *Panonychus citri***. Click here for file

Additional file 2 **Comparisons of mitochondrial genome organizations of *Panonychus citri*, *P. ulmi *and *Tetranychus urticae***. Click here for file

Additional file 3 **Mitochondrial genome arrangements of 26 Acari**. Only protein coding genes (PCGs) and ribosomal RNA genes (rRNAs) are given, whereas transfer RNA genes (tRNAs) are not depicted because they are highly complex. White boxes represent genes with the same relative position as in the arthropod ground pattern, *Limulus polyphemus*. Blue color indicates translocations, only, whereas yellow color denotes translocations and inversions, combined. All genes are transcribed from left to right except those underlined to indicate an opposite transcriptional orientation. Click here for file

Additional file 4 **Base composition at each codon position of the concatenated 13 PCGs in the Acari mitochondrial genomes**. See Figure 2 for the full names of species. Click here for file

Additional file 5 **Amino acid usage of mitochondrial protein-coding genes from other Acari**. Click here for file

Additional file 6 **The correlation between the four most frequently used codons and overall A + T content in the Acari mitochondrial genomes**. Click here for file

Additional file 7 **Alignment of the sequences of the 22 mitochondrial tRNA genes of *Panonychus citri*, *P. ulmi *and *Tetranychus urticae***. Pc-Y is *Panonychus citri *in this study; Pc-L is another *P. citri *strain (GenBank: NC_014347); Pu is *P. ulmi*; Tu is *Tetranychus urticae*. The anticodon arms are indicated with underlines. Click here for file

Additional file 8 **The inferred secondary structures of tRNA genes of another *Panonychus citri *strain, *P. ulmi*, and *Tetranychus urticae***. The added nucleotides are indicated in red color, whereas the deleted nucleotides are underlined, compared to the original annotations of tRNA genes on GenBank. Pc-L is another *P. citri *strain (GenBank: NC_014347). Click here for file

Additional file 9 **Average size of tRNA genes of 26 Acari mitochondrial genomes**. The Acariformes are indicated by red color and the Parasitiformes by blue color. See Figure 2 for the full names of species. Click here for file

Additional file 10 **Primer sequences and PCR conditions used to amplify the mitochondrial genome of *Panonychus citri***. Click here for file

Additional file 11 **GenBank accession numbers of mitochondrial genomes for other Acari and the horseshoe crab *Limulus polyphemus***. Click here for file
