# Supplementary material for: The complete mitochondrial genome of the citrus red mite Panonychus citri (Acari: Tetranychidae): high genome rearrangement and extremely truncated tRNAs
Source: BMC Genomics. 2010 Oct 23;11:597. doi: 10.1186/1471-2164-11-597 (PMC3091742; doi:10.1186/1471-2164-11-597)
Supplement: Additional file 1 — Summary of the mitochondrial genome of Panonychus citri. [file 1471-2164-11-597-S1.DOC]

Additional File 1 - Summary of the mitochondrial genome of *Panonychus citri*

| Gene^a^ | Position | Size | inc^b^ | AT% | AT-skew^c^ | AT/4^d^ | GC-skew^c^ | GC/4^d^ | fcd^e^ | scd^e^ | acd^e^ |
| --- | --- | --- | --- | --- | --- | --- | --- | --- | --- | --- | --- |
| *cox1* | 1-1536 | 1536 | 0 | 78.45 | -0.162 | -0.122 | 0.003 | 0.200 | ATC | TAA |  |
| D-loop | 1537-1593 | 57 | 0 | 100.00 |  |  |  |  |  |  |  |
| *nad3* | 1594-1914 | 321 | 0 | 89.72 | -0.243 | -0.600 | 0.030 | -1.000 | ATA | TAA |  |
| *trnN* | 1916-1975 | 60 | 1 | 77.42 | 0.000 |  | 0.429 |  |  |  | GTT |
| *trnD* | 1977-2029 | 53 | 1 | 88.68 | 0.064 |  | 0.333 |  |  |  | GTC |
| *trnL1* | 2033-2084 | 52 | 3 | 87.50 | 0.102 |  | 0.429 |  |  |  | TAG |
| *trnE* | 2085-2134 | 50 | 0 | 82.00 | -0.073 |  | -0.333 |  |  |  | TTC |
| *rrnL* | 2135-3123 | 989 | 0 | 86.66 | -0.006 |  | 0.145 |  |  |  |  |
| *trnR* | 3124-3174 | 51 | 0 | 84.62 | -0.091 |  | 0.000 |  |  |  | TCG |
| *nad4L* | 3185-3433 | 249 | 10 | 89.56 | -0.157 | -0.091 | 0.077 | -1.000 | ATA | TAA |  |
| *trnP* | 3431-3481 | 51 | -3 | 84.44 | 0.000 |  | 0.143 |  |  |  | TGG |
| *trnF* | 3479-3535 | 57 | -3 | 84.21 | -0.042 |  | 0.111 |  |  |  | GAA |
| *cox3* | 3536-4322 | 787 | 0 | 83.10 | -0.165 | -0.123 | -0.008 | -0.667 | ATG | T |  |
| *atp6* | 4326-4946 | 621 | 3 | 86.63 | -0.123 | 0.087 | -0.229 | -0.333 | ATG | TAA |  |
| *atp8* | 4947-5073 | 127 | 0 | 87.40 | -0.117 | -0.714 | 0.000 | -1.000 | ATT | T |  |
| *trnK* | 5074-5137 | 64 | 0 | 70.31 | -0.022 |  | -0.053 |  |  |  | CTT |
| *cox2* | 5148-5777 | 630 | 10 | 81.90 | 0.000 | -0.061 | -0.018 | 0.200 | ATG | TAA |  |
| *trnY* | 5783-5830 | 48 | 5 | 92.45 | -0.102 |  | 0.000 |  |  |  | GTA |
| *rrnS* | 5831-6478 | 648 | 0 | 87.54 | 0.104 |  | 0.160 |  |  |  |  |
| *trnG* | 6479-6537 | 59 | 0 | 77.78 | 0.238 |  | -0.500 |  |  |  | TCC |
| *trnT* | 6538-6594 | 57 | 0 | 89.47 | -0.020 |  | 0.333 |  |  |  | TGT |
| *nad1* | 6597-7452 | 856 | 2 | 84.93 | -0.164 | 0.000 | -0.008 | -0.600 | ATA | T |  |
| *trnL2* | 7453-7517 | 65 | 0 | 83.08 | 0.111 |  | -0.091 |  |  |  | TAA |
| *trnQ* | 7505-7555 | 51 | -13 | 83.67 | -0.220 |  | 0.000 |  |  |  | TTG |
| *trnC* | 7551-7606 | 56 | -5 | 78.57 | -0.045 |  | -0.167 |  |  |  | GCA |
| *cob* | 7613-8677 | 1065 | 6 | 81.69 | -0.230 | -0.451 | 0.026 | 0.250 | ATT | TAG |  |
| *trnS2* | 8676-8719 | 44 | -2 | 87.04 | 0.021 |  | 0.429 |  |  |  | TGA |
| *trnA* | 8718-8769 | 52 | -2 | 84.09 | 0.081 |  | 0.429 |  |  |  | TGC |
| *nad6* | 8760-9162 | 403 | -10 | 92.06 | -0.197 | -0.444 | -0.063 | 1.000 | ATT | T |  |
| *nad4* | 9163-10323 | 1161 | 0 | 86.39 | -0.230 | -0.325 | 0.114 | 0.200 | ATG | TAA |  |
| *trnH* | 10323-10378 | 56 | -1 | 83.33 | -0.022 |  | 0.333 |  |  |  | GTG |
| *nad5* | 10379-11930 | 1552 | 0 | 87.50 | -0.166 | -0.170 | -0.010 | 0.333 | ATT | T |  |
| *trnW* | 11931-11984 | 54 | 0 | 88.89 | 0.083 |  | 0.000 |  |  |  | TCA |
| *nad2* | 11985-12872 | 888 | 0 | 91.33 | -0.253 | -0.500 | -0.091 | 0.000 | ATT | TAA |  |
| *trnM* | 12889-12943 | 55 | 16 | 69.09 | -0.158 |  | -0.059 |  |  |  | CAT |
| *trnS1* | 12943-12989 | 47 | -1 | 81.40 | -0.086 |  | -0.250 |  |  |  | GCT |
| *trnV* | 12989-13037 | 49 | -1 | 88.37 | 0.211 |  | -0.600 |  |  |  | TAC |
| *trnI* | 13024-13077 | 54 | -14 | 88.64 | -0.128 |  | 0.200 |  |  |  | GAT |

^a^ genes coded in N-strand are underlined. ^b^ inc = intergenic nucleotides, indicates gap nucleotides (positive value) or overlapped nucleotides (negative value) between two adjacent genes. ^c^ AT-skew = (A-T)/(A+T), GC-skew = (G-C)/(G+C). ^d^ skews at 4-fold degenerated sites. ^e^ fcd, first codon; scd, stop codon; acd, anticodon.
