# Supplementary figures and images for: The complete mitochondrial genome of the citrus red mite Panonychus citri (Acari: Tetranychidae): high genome rearrangement and extremely truncated tRNAs
Source: BMC Genomics. 2010 Oct 23;11:597. doi: 10.1186/1471-2164-11-597 (PMC3091742; doi:10.1186/1471-2164-11-597)

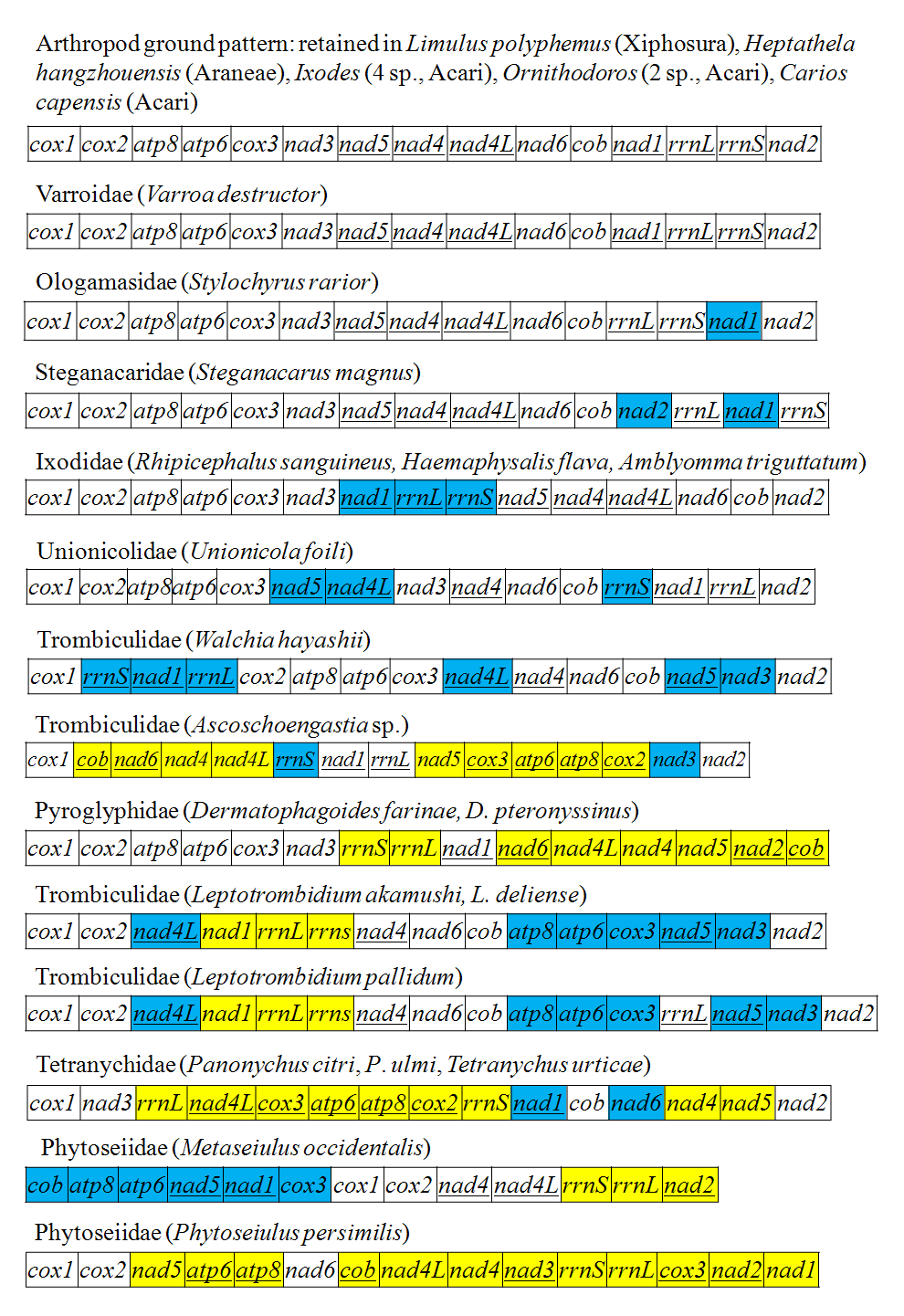

Supplement: Additional file 3 — Mitochondrial genome arrangements of 26 Acari. Only protein coding genes (PCGs) and ribosomal RNA genes (rRNAs) are given, whereas transfer RNA genes (tRNAs) are not depicted because they are highly complex. White boxes represent genes with the same relative position as in the arthropod ground pattern, Limulus polyphemus. Blue color indicates translocations, only, whereas yellow color denotes translocations and inversions, combined. All genes are transcribed from left to right except those underlined to indicate an opposite transcriptional orientation. [file 1471-2164-11-597-S3.TIFF]

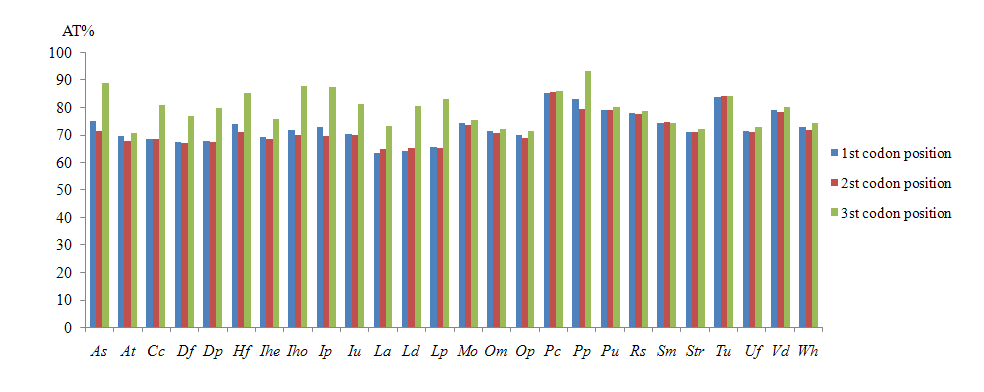

Supplement: Additional file 4 — Base composition at each codon position of the concatenated 13 PCGs in the Acari mitochondrial genomes. See Figure 2 for the full names of species. [file 1471-2164-11-597-S4.TIFF]

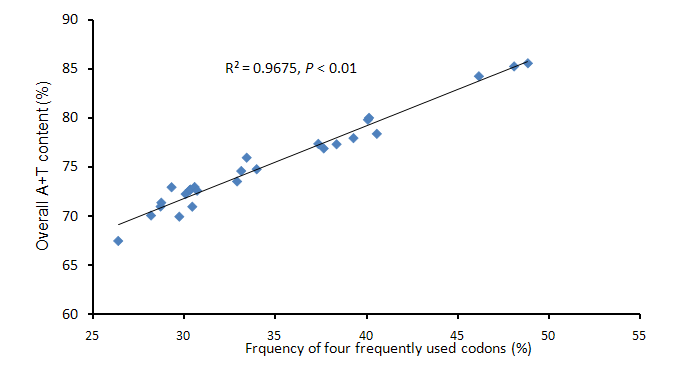

Supplement: Additional file 6 — The correlation between the four most frequently used codons and overall A + T content in the Acari mitochondrial genomes. [file 1471-2164-11-597-S6.TIFF]

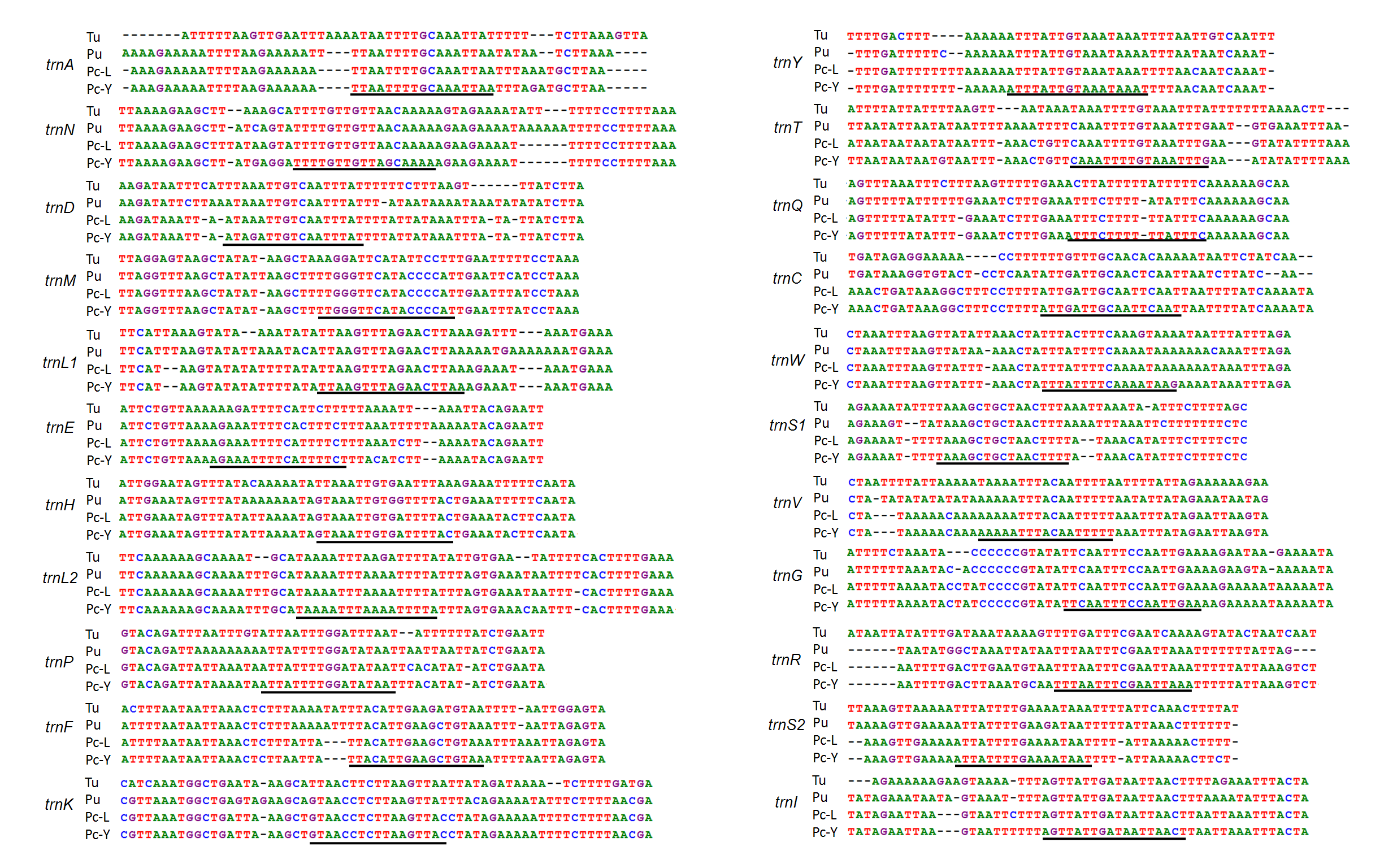

Supplement: Additional file 7 — Alignment of the sequences of the 22 mitochondrial tRNA genes of Panonychus citri, P. ulmi and Tetranychus urticae. Pc-Y is Panonychus citri in this study; Pc-L is another P. citri strain (GenBank: NC_014347); Pu is P. ulmi; Tu is Tetranychus urticae. The anticodon arms are indicated with underlines. [file 1471-2164-11-597-S7.TIFF]

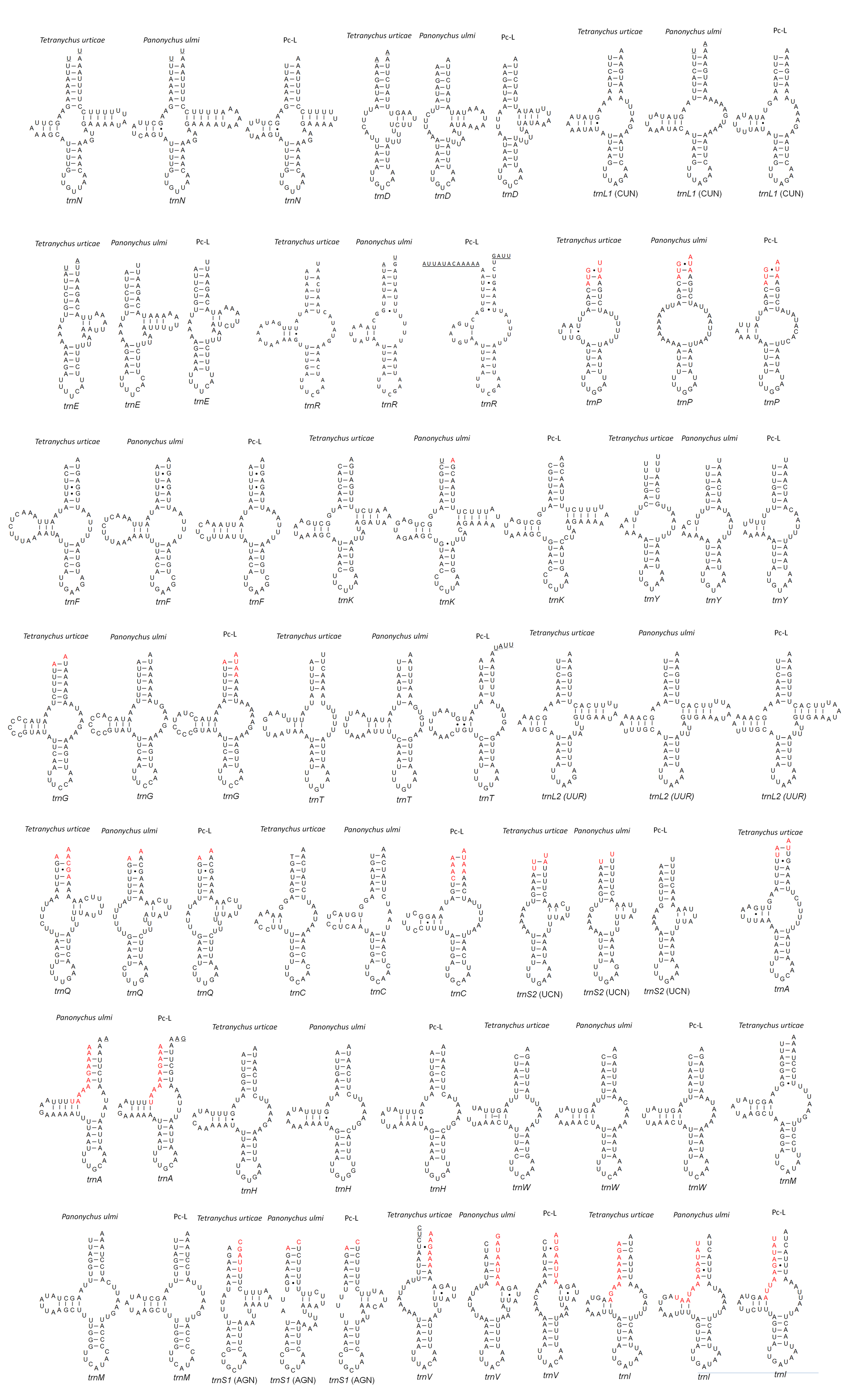

Supplement: Additional file 8 — The inferred secondary structures of tRNA genes of another Panonychus citri strain, P. ulmi, and Tetranychus urticae. The added nucleotides are indicated in red color, whereas the deleted nucleotides are underlined, compared to the original annotations of tRNA genes on GenBank. Pc-L is another P. citri strain (GenBank: NC_014347). [file 1471-2164-11-597-S8.TIFF]

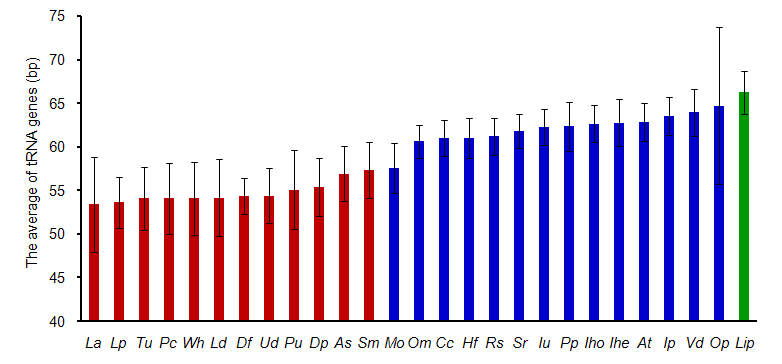

Supplement: Additional file 9 — Average size of tRNA genes of 26 Acari mitochondrial genomes. The Acariformes are indicated by red color and the Parasitiformes by blue color. See Figure 2 for the full names of species. [file 1471-2164-11-597-S9.TIFF]
