# Supplementary material for: The complete mitochondrial genome of the citrus red mite Panonychus citri (Acari: Tetranychidae): high genome rearrangement and extremely truncated tRNAs
Source: BMC Genomics. 2010 Oct 23;11:597. doi: 10.1186/1471-2164-11-597 (PMC3091742; doi:10.1186/1471-2164-11-597)
Supplement: Additional file 11 — GenBank accession numbers of mitochondrial genomes for other Acari and the horseshoe crab Limulus polyphemus. [file 1471-2164-11-597-S11.DOC]

| **Species** | **Classification** | **GenBank accession number** |
| --- | --- | --- |
| *Amblyomma triguttatum* | Arachnida, Acari, Parasitiformes | NC_005963 |
| *Ascoschoengastia* sp. | Arachnida, Acari, Acariformes | NC_010596 |
| *Carios capensis* | Arachnida, Acari, Parasitiformes | NC_005291 |
| *Dermatophagoides farinae* | Arachnida, Acari, Acariformes | NC_013184 |
| *Dermatophagoides pteronyssinus* | Arachnida, Acari, Acariformes | NC_012218 |
| *Haemaphysalis flava* | Arachnida, Acari, Parasitiformes | NC_005292 |
| *Ixodes hexagonus* | Arachnida, Acari, Parasitiformes | NC_002010 |
| *Ixodes holocyclus* | Arachnida, Acari, Parasitiformes | NC_005293 |
| *Ixodes persulcatus* | Arachnida, Acari, Parasitiformes | NC_004370 |
| *Ixodes uriae* | Arachnida, Acari, Parasitiformes | NC_006078 |
| *Leptotrombidium akamushi* | Arachnida, Acari, Acariformes | NC_007601 |
| *Leptotrombidium deliense* | Arachnida, Acari, Acariformes | NC_007600 |
| *Leptotrombidium pallidum* | Arachnida, Acari, Acariformes | NC_007177 |
| *Metaseiulus occidentalis* | Arachnida, Acari, Parasitiformes | NC_009093 |
| *Ornithodoros moubata* | Arachnida, Acari, Parasitiformes | NC_004357 |
| *Ornithodoros porcinus* | Arachnida, Acari, Parasitiformes | NC_005820 |
| *Panonychus ulmi* | Arachnida, Acari, Acariformes | NC_012571 |
| *Phytoseiulus persimilis* | Arachnida, Acari, Parasitiformes | NC_014049 |
| *Rhipicephalus sanguineus* | Arachnida, Acari, Parasitiformes | NC_002074 |
| *Steganacarus magnus* | Arachnida, Acari, Acariformes | NC_011574 |
| *Stylochyrus rarior* | Arachnida, Acari, Parasitiformes | NC_013474 |
| *Tetranychus urticae* | Arachnida, Acari, Acariformes | NC_010526 |
| *Unionicola foili* | Arachnida, Acari, Acariformes | NC_011036 |
| *Varroa destructor* | Arachnida, Acari, Parasitiformes | NC_004454 |
| *Walchia hayashii* | Arachnida, Acari, Acariformes | NC_010595 |
| *Limulus polyphemus* | Merostomata, Xiphosura, Limulidae | NC_003057 |
